# Reviews of Dental Literature

**Published:** 1905-04

**Authors:** 


### Reviews of Dental Literature.

In the *British Dental Journal* for September, 1904, is a paper on "Vulcanite Crowns," by E. R. Tebbitt, L.D.S.

These are used for trial crowns, and for the first permanent molars of children between the ages of eight and fourteen, and lower molars which are beyond crowning by the usual methods, as where the root presents a saucer-shaped depression on the gum level, the edges soft and the remaining dentine hard. After preparation of the canals, clear out the general surface of the root with large, coarse burs. If the gum is overhanging, a copper tack with a good supply of gutta-percha will leave a fair field after being in place three days. A gold pin, the projecting end of which has been well flattened, is fitted in the canal, or where the best masticating surface is wanted, and if it is not necessary to avoid the jar use a platinum pin, spread the top with a riveting hammer, and adapt it to take the brunt of the bite. The author has not found a second pin necessary. After the pin is fitted an impression and bite are taken. Cast in Spence metal and mount on an articulator. Remove the pin and oil the root and surrounding gum with any viscid oil. Replace the pin and drop melted wax around it to just cover the root, and if a porcelain facing is to be used it should be fitted; continue to build up to the bite with wax, trimming the edges so that no part of the wax overlaps the gum. Invest, exposing the lingual aspect so as not to disturb either the bite or fit of the crown; as the elasticity of the rubber is of no consequence, but hardness everything, it is well but not necessary to give it two hours in the vulcanizer.

Spence metal is composed of sixty parts of sulphide of iron and forty parts of sulphur. The fusing point is 225° F. It is of great value when used as an articulating model in setting up an upper or lower denture, by minimizing the constant attrition of the antagonistic surfaces, which more or less, according to the ability of the mechanic, takes place when teeth of porcelain are brought into repeated contact with very much softer plaster ones. For all models, therefore, to be used solely for antagonizing porcelain teeth during the setting-up process, one will find that Spence metal will not show any signs of wear on the cusps, and that, other things being equal, the case when in the mouth will present the same occlusion as on the articulator.

Used in casting models for metal work, again it has distinct advantages over plaster. Think for a minute of the result of trying an imperfectly fitting gold plate on a plaster model. It is more or less gently rocked to see where it bears heavily, at the same time it rubs an easement for itself where it does not fit; and this process is perhaps repeated after the plate has been swaged once more, with the result that it seems to settle down to reasonable fit. Let the model be of Spence metal, and the least rock is at once apparent, and no amount of persuasive wriggling will wear the model to fit the plate.

It is often difficult to cast a model in Spence metal sufficiently deep to get a reasonably heavy zinc from it. The simplest solution of this difficulty is to cast the Spence metal so as to cover the highest part of the impression material by about a quarter of an inch, then watch for the first signs of setting on the lower edge of the cast, and when the rim has solidified about a quarter of an inch, pour out the remaining molten metal, and the result will be a hollow cast with an inward turned rim. This is now easily filled with plaster and built up to the required height, and the rim will hold the two together quite securely.

For models to be used in fitting a cap crown it has this good point: After fitting the collar to the root, many operators take an impression of the banded tooth with the band in position, and cast the whole in plaster; when the band is removed from the plaster cast, it not infrequently brings with it that part of the model which represents the root, leaving only a fine mark to indicate the correct position. If the impression taken in plaster or composition is oiled and cast in Spence metal, the band may be taken on or off at will, and we may be sure that any slight accidental bend it may get will be corrected when it is next placed on the model.

Like a plaster model, the best result is obtained from a plaster impression; it is nevertheless easily used with other impression materials, ordinary Stent's being very satisfactory, provided reasonable care is taken to pour the metal at the right moment and to direct the stream into some unimportant part of the impression. The Stent should be well chilled before pouring the Spence metal.

For those who did not know the method of manipulating this substance he outlined the way in which it was handled. As bought, it is a grayish-black powder, and is melted in an ordinary enamel saucepan, most conveniently over a Bunsen burner, the flame of which must not be allowed to pass up the sides of the pan, for if it gets on the thin layer of metal adhering to the sides of the pan, the metal will be overheated and give off strong sulphur fumes, and at the same time considerably deteriorate. The impression to be cast is preferably surrounded by one of the "rapid casting cups," and is previously oiled with one of the heavier cycle lubricating oils.

On first applying the heat, when the metal is in powder, it is necessary to stir it until a little of it is melted at the bottom of the pan. The flame is then reduced and the pan shaken over it till the whole forms a very awkward-looking mass, being a kind of stiff black froth. The pan should be now removed from the flame and the contents stirred with a piece of stick. This stirring, with the occasional application of further gentle heat, will reduce the whole into an extremely fluid condition, and when the setting (which begins at the higher parts of the pan) has nearly reached the general level of the liquid portion the metal is poured rapidly, directing the stream on the middle of the back edge of the impression. As soon as set, which takes from one to two minutes, according to the bulk of the metal, the rubber cup is removed.

When casting into a plaster impression it is best to let the whole cool before the plaster is removed, considerably less delicacy being necessary than where plaster is used for both impression and model. If composition has been the impression material, as soon as the rubber cup is detached gentle traction may be exerted on the heel of the composition, when the contained heat of the metal will soften it sufficiently to allow of its removal.

A difficulty, which is only a temporary one, is that when first working on black models after being used to white, the whiter porcelain tooth seems to stand out between its darker neighbors, and the tendency is to set it too far in.

If the material becomes "stodgy," a small addition of sulphur will restore its efficiency.

Comparing Spence metal and plaster, the former is harder, cleaner, takes less time from mouth to finished model, and can be used over and over again.

Spence metal can be procured of C. Ash & Sons.

In the *British Dental Journal* for November there appears a communication by Harry Baldwin, M.R.C.S., L.D.S., entitled "Further Experience of Cement and Amalgam Fillings."

The author advocates a method of filling teeth by lining the cavity with oxyphosphate of zinc, placing amalgam into the cement while still soft, and finishing with an amalgam surface. The points in favor of this method were, that it required a much smaller sacrifice of healthy tooth substance, leaving a stronger tooth less liable to subsequent fracture and necessitating much less pain in excavating. It interposes a non-conducting layer between the sensitive dentine and the metal. Compared with amalgam it does not stain the tooth nor show a nasty color through a thin enamel wall, is more water-tight, and adheres to the cavity.

The author has used this method for a number of patients who presented themselves originally with all or most of their back teeth ragged and ruinous, containing the eroded and abraded remains of phosphate of zinc fillings, presenting everywhere rough edges, spaces into which food crowded, softening edges of exposed dentine, and such an inefficient masticatory mechanism as to be lamentable. These cases now present smooth, well-filled shapes, with restoration of original contours, abolition of irritating spaces, good sound metallic surfaces in contact, and in a condition which may be described as permanent. These cases are very satisfactory, not only because of the improved condition but also because of the extreme simplicity of the means by which the result was secured.

As the beauty of the combination method depends entirely upon a strong and effective adhesion, it is not necessary to emphasize the importance of using materials that are favorable. The best cement, as far as Dr. Baldwin knows, is "Harvard," and the best amalgam, "Standard" (Eckfeldt & Dubois).

In order to get the best results the amalgam should be packed in a manner not specially inviting shrinkage or change of shape, and the best principle on which to work is that of Bonwill. Bonwill's method consists in forcibly squeezing the amalgam, after packing it in the tooth, with a pad over its whole free surface simultaneously. The pad may be of any soft material, but rolled up thin bibulous paper and india-rubber are the two most generally used. The practical way of effecting this entire compression of the amalgam in the cavity of a tooth is to use a matrix wherever possible, and to squeeze powerfully over the whole free surface of the amalgam with a closely fitted pad. The mercury which is in excess in the amalgam will be forced out, and will escape from under the edges of the pad in fine bright drops, or can be easily scraped away with a spoon excavator on removal of the pad. The greatest care must be taken to pack the amalgam with a fine, almost pointed plugger in the retiring angles between the matrix and the cavity edges, great pressure being used in these positions so as to insure the amalgam being well packed, as dense and as free from surplus mercury as the central portions; otherwise these portions will receive less pressure than the rest, and will absorb surplus mercury from the rest, and so give bad shrinking edges to the filling. The reason for eliminating the surplus mercury is twofold,—first, to make a denser and harder filling; secondly, to prevent change of shape, warpage, in the finished mass.

To understand something of the nature and behavior of amalgam, one should realize that a soft amalgam is in the condition of a sponge containing a quantity of free mercury between its interlacing fibres, just as a sponge contains water in its interspaces.

A good deal of misconception regarding the reason for, and manner of, change of shape in a setting amalgam or amalgam after setting seems still to exist. Authors will be found alluding to the tendency of amalgam to "ball," just as if amalgam were a fluid, like water or mercury, to take a spheroidal shape.

A stiff paste like a setting amalgam has no surface tension and cannot attempt to ball. What happens with a warping amalgam, when that amalgam is of good quality and compounded so as not to shrink or expand, is that, owing to an uneven distribution of the mercury, the mercury is proceeding to redistribute itself more evenly, those parts of the mass which contain less mercury absorbing it from those parts which contain more, and causing an expansion of those parts receiving mercury and a contraction of those parts giving it up. The reason for an amalgam filling, packed merely with steel instruments, containing an uneven distribution of mercury, is simply that the deeper portions are well compressed by the superincumbent layers, and so have had their surplus mercury squeezed out of them, and this surplus has been absorbed by the outer layers. The outermost layer then contains not only its own excess, but a surcharge absorbed from the layers beneath.

Pressure with a steel instrument merely displaces the excess from immediately under the face of the instrument, and the surrounding surface of amalgam simultaneously absorbs it.

It will readily be seen that expression by pressure applied over the whole surface is the best method of getting rid of the surplus, because any method of absorbing it by tin or silver cylinders, unless accompanied or followed by great and well-adapted pressure, will induce shrinkage of the outer part and a porous surface.

PROFESSIONAL EMANCIPATION AND HOW IT MAY BE EFFECTED.[1] By J. E. Weirick, D.D.S., St. Paul, Minnesota.

It is with a deep sense of appreciation that I am taking advantage of your kind invitation to deliver an address at this meeting. If I had my just deserts, I would be begging at the back

[1] Read before the Indiana State Dental Society, June, 1904.

door of your hall for permission to appear here in the rôle of a penitent. That I am, instead, honored with the freedom of the floor and the privileges of a speaker denotes your generous spirit, and is another demonstration of that fine broad character for which Indianapolis and her people have long been famous among those who have enjoyed the boon of your friendship and hospitality. But even *your* indulgence must have been tested in my case. For I plead guilty to having outraged your kindness last year when, after accepting an invitation to attend the annual meeting here, I failed at the last minute and that without warning or advance notice. I know of nothing that has come to me so pleasantly as the renewal of your invitation this year, showing that you had fully forgiven my trespass. I will not try your patience with an explanation of my non-appearance at the last convention, except to say that it was caused by an entirely unexpected professional matter, that pressed on me so urgently I could not get away from St. Paul, though you may rest assured I would have hesitated at no possible sacrifice to fill my engagement with you. Instead, then, of offering a long explanation, I will try to show my appreciation of your forbearance by making my address here as short and sharp and pointed as possible; wasting as little of your time as may be. If you find some of the things I say blunt and undiplomatic and crudely put, please ascribe it to the desire on my part to use as few words as possible. And this request leads naturally to the main idea on which I have taken the liberty to base my address,—that we dentists at our meetings and conventions are entirely too prone to waste our time in bootless talk and impractical action; that we generally come to these gatherings with a bag of wind under one arm and a keg of water under the other; varying the programme only occasionally, by carrying something other than water in the keg.

True, we are no greater sinners in this respect than many of our fellows in the other professions. But there is this difference: They can afford to indulge in a harvest of wind; we cannot. Their status is established; ours is not. They have a professional ancestry; we, as yet, have only a professional progeny. They stand on a solid foundation built for them by their pioneers; we have our foundation still to build, or at least to make solid; and we are not doing it. Instead, we are drifting along in a haphazard way, deceived by the crumbs of recognition thrown us here and there into the belief that we have really arrived; that we are really and broadly ranked with the other learned professions. As a matter of fact, we are not. The more clearly we understand this, the better. For, with such an understanding will come properly directed work, concerted effort, and the measure of achievement that will give us the *substance* of that *high standing;* of which we have now but the *shadow.* At least, I believe that this will be so; that we have but to make clear to ourselves and our associates the ambiguous position we occupy, in order to bring about a condition of affairs that will make any man proud of the fact that he belongs to this profession. Indeed, I believe it only necessary that we wake up to our true situation, to insure our becoming leaders instead of followers in the world's division of medicine and healing. We have but to look back over the history of our progress so far, to make very clear the potential possibilities of our craft. Hampered by the most serious disabilities, disabilities that I will refer to in detail with your permission, we have yet to record the most extraordinary progress. Let us overcome these disabilities, and we will at once take proper rank, and win that professional standing which it must be the aim and desire of every properly constituted man to have for his own.

It is true, undoubtedly, that, even with the progress we are making to-day, we will eventually arrive. The things that we have done demonstrate this clearly enough; but they demonstrate, also, that we are still far from the goal, and that as matters are now moving, it will be a generation, or even two generations, before we can fairly say that our profession is abreast in rank and consideration with the other sections of medical science. And for myself, that rate of progress is too slow. I am not content with the knowledge that the next generation of American dentists, or the one after will come into full recognition. I want this generation to see such a result.

I am tired of seeing the Section on Stomatology of the American Medical Association tagging perpetually at the rear end of the procession. I want to see this section on an equality in the councils of the Association with the sections on ophthalmology, surgery, and the others. I want to see an end to the condescension and the patronizing that are always extended to the practitioners in our profession by the members of the older branches of medicine.

It is unnecessary to point out to any one who has attended the meetings of the American Medical Association the *humiliating position* that we always occupy. In our personal capacity, we are well enough. We have nothing to complain of at the social functions. But when it comes to a voice in the direction of affairs scientific, we find ourselves barely tolerated.

It is my firm belief that the work we are doing is as important as is the work in any other division of medicine. In numbers we are stronger than any of the other sections. There are to-day in the United States thirty-five thousand registered dentists. No other division of specialists can show within twenty thousand of that number. We have made relatively greater progress than any other branch. We have undoubtedly done more to ameliorate suffering than any equal number of men in the world. We play a most important part in the physical upbuilding of the race, by supplying and maintaining sound machinery for the mastication and consequently for the digestion of food. We have added more to the beauty of the human countenance, during the few years we have been at work, than have all the other agencies in the world before us.

Why, then, with such a record, do we still find ourselves at the foot of the ladder among the professions? The answer is to be found in ourselves; in the lack of properly directed effort, or rather in the lack of thoughtful effort. We are started wrong from the moment we leave college, almost from the moment we enter college, and somehow we never do seem to get right. The taint of commercialism is on us from the very beginning, a *small, pitiful, petty* commercialism. We are yoked at the outset of our careers to a chariot that carries a company of mercenaries, and very few of us ever develop sufficient strength to throw off the yoke. Nor is this to be wondered at in the circumstances.

We all know that the boys who go into dental colleges are, for the most part, poor. They come from the farms, out of village stores, out of shops in the large cities, and, in a few instances, very few, out of colleges, with just about enough money to see them through the three or four years' course. They are almost always dependent on their own resources, and where their parents or relatives are in a position to help, they provide only sufficient funds to see them through to graduation. From that point the young fellow has his own way to make.

It may very properly be argued that a similar situation prevails in the other professions. But at the point of graduation there is this difference: The young medical student or lawyer goes out under healthy, normal conditions. The young dental student is met at the threshold of his professional life by a band of men, who make it their business to put him at once in a mortgaged condition. I refer, as you no doubt are aware, to the agents of the dental supply houses. Under the present easy-going system, these agents are permitted to mingle freely with the students inside the college walls, to win their friendship and confidence, and to put themselves generally in a position where they may, at graduation, work the young man just as they please. And what is the result? The graduates are tempted into a business deal that leaves them for years, if not throughout their lives, hopelessly in debt. They are beguiled into buying equipment and supplies utterly beyond their means, and far in excess of their necessities. And *there* is laid the foundation of the evil condition that afflicts the entire profession. It is the beginning of the commercial domination that I believe to be the main source of our inability to rise to a level with the other professions. The college authorities, instead of combating this situation, foster it, not because of any selfish or mercenary motive, but because of habit. It has always been so.

The supply people are clever, capable fellows, generally personal friends of the best men in the faculty, and no one dreams of interfering with them. It was the way when I went to college, and it will be the way when the generation still unborn go, unless we get together and put a stop to it, arousing the interest of the college officials, and making clear to them the need for reform. It is hopeless to look for relief to the supply people. With them it is business; and good business. Experience has taught them that it is a perfectly safe proceeding to trust every graduate who may be induced to buy. It is the same scheme that is worked in another form in the mining districts of Pennsylvania, in the plantation districts of the South, and in the milling districts of other States, where the "company store" grants unlimited credit, knowing full well that it has absolute security in the wages to be earned.

In no profession, except ours, does such a situation exist. I can quite conceive of what would happen to any firm, or corporation, or combination, that should attempt systematically to load down the graduate of a medical college with an unnecessary and uncalled for burden of debt, that should attempt to impose upon the inexperience and credulity of young men utterly incapable of judging for themselves of the conditions they will have to face in the working world. But in the dental colleges usage has not alone made this condition a matter of course, but the system has its beginning almost at the outset of the young man's career as a student.

It is my firm belief that the present unsatisfactory standing of the profession is, to a large extent, due to this false start. If the students we send out of our colleges were protected against loading themselves down with a wholly unnecessary outfit, for which they mortgage at least the beginning of their careers, matters would soon right themselves and that taint of commercialism, that to my mind is the greatest curse and the greatest handicap of the profession, would soon disappear.

As it is, the young dentist starts out in life as the bond-slave of the man or combination that has sold him his outfit, and he spends a large part of his professional career in an endeavor to get out of debt. For the most part this is a slow, up-hill struggle. In many instances it is a *hopeless* struggle, and the man begins and ends in debt. In any event, the task hardens the victim and obscures, if it does not wipe out entirely, the humanitarian side of his nature. He looks upon his profession simply as a *money-making* machine, and he sells his services just as the grocer sells sugar and potatoes, and the butcher sells beef.

Only in exceptional cases, and with exceptional men, does a love of the profession survive this situation. For the most part the dentists gradually become merely distributing agents for the supply dealers and manufacturers, bent on getting as large a percentage as possible for their services in this direction. They become tradesmen in spirit, if not in fact, and the pitiful thing is that they must ever remain petty tradesmen, getting nothing like their just due, no matter how mercenary their spirits.

This condition crushes out the broad spirit that should characterize a true representative profession, and that does characterize most professions. The young physician and the young lawyer, whatever their hardships at the start or throughout life, realize, at least, that they are working for themselves and not for a master who holds a bond on their implements of trade, and who controls every avenue of their activity. This gives them time and inclination to look to higher and better things. *They* are not forever faced with the pecuniary and commercial side of their calling. They find themselves in a profession that is free and untrammelled and not dominated from beginning to the end, from roots to branches, by a commercial oligarchy.

We, who have become hardened to this condition, inured to the atmosphere, may not fully appreciate what an evil effect this commercial despotism has on us. We may not appreciate how far it goes toward making the difference in grade that lies between other professions and ours. But it is only necessary to examine calmly into the facts to arrive at a most convincing conclusion.

I suppose you all know that the great barbers' supply houses and the big breweries have a system that is very much akin to that of the dental supply houses. They find likely men with a little money and fix up for them establishments more or less elaborate, upon which they take a mortgage. And thereafter the barber and the saloon-keeper are simply distributing agents for their masters, just as are the dentists for theirs, unless they have sufficient talent or shrewdness to pay themselves out of their clutches.

I confess that it is rather humiliating to find such a comparison in order. But the facts force it upon us. And, further, the facts show that this situation is by no means confined to the time of graduation, to the time when we enter our profession. So habituated do we become to the domination of the supply men, that we carry them with use in happy comradeship throughout our careers.

In other professions, the men who make and sell the necessary tools, and adjuncts, one meets at their offices or they call at stated intervals to offer and push their wares. But with us they are, at the beginning and at the end, the main thing. At our important conventions the choicest rooms and the most desirable quarters are pre-empted by the supply houses. The agents hobnob with us, and thrust their hospitality upon us, and we are, apparently, glad to have them do so. Can you imagine a medical convention or a lawyers' convention manipulated or controlled by implement and supply men?

I am aware that at the conventions of trade and commercial bodies the manufacturers' displays form one of the most important features. But we are forever boasting that we are far and away above trade and commercialism. We are forever striving for the elevation of our profession and for higher standards of dignity. Yet, wherever we meet in considerable numbers, we are fairly inundated with trade displays. The only difference in this respect between us and the trade convention is that where, for example, the hardware men are confronted with ice-cream freezers, snow-shovels, and patent road-scrapers, and the plumbers' rooms are furnished with the newest styles of sanitary plumbing, we meet chisels, excavators, patent cuspidors, and other cheerful instruments. Mind, I am not finding fault on personal grounds, either with the supply houses or their agents. I know many charming gentlemen and fine fellows in the business, men whom I am glad to meet at all proper times and on all proper occasions, as my friends. Men whom I am glad to have at my home and to whom I am happy to extend hospitality. But I do object to the spirit that it breeds, to the domination of our conventions and our actions by men who, if we asserted ourselves and valued ourselves as do other professions, would be very glad to remain in the background on these occasions when we are supposed to meet for the interchange of experience and opinion. The whole system should be torn up, root and branch, and must be before we can attain to the dignity and standing after which we are all so ardently striving.

A striking manifestation of what we are coming to, or rather what we *have* come to under this system of commercial control, is to be found in our literature. Look the field over, and with one exception you do not find a dental journal that is not controlled by a supply house. And *brazenly* controlled. *No effort* is made at concealment. *There, on the cover of each paper,* you will find calmly set out that it is published by this supply house or that. And the editors are among the best known men in the profession. *This is a condition that is not alone without parallel in the professions, but without parallel in trade.* I have mentioned this peculiarity to a number of friends who are engaged in trade journalism, and without exception they were filled with amazement.

That we should have *not one widely read independent* influential paper of tone, dignity, and national standing, seemed to them an *impossible* situation. Yet, I confess, on me this anomalous condition had never made impress until I began to investigate it. And so, no doubt, it is with you. We have become so habituated to the existing condition, that the possibility of anything better, or different, has probably never suggested itself to us. All the other professions have a number of high-grade independent periodical publications to represent them. So far as I have been able to find, there is no trade *so lowly* that has not its independent mouthpiece. Only the *dental profession* must depend for its periodical literature on "house organs." Probably you do not know what a "house organ" is. It is a technical term applied in trade to publications issued in the interest of an individual firm or house, and such organs are looked on with scorn by the members of the trade. They have neither weight nor influence, but are frankly advertising devices. In the professions, other than ours, such publications are entirely unknown, or if they exist they have no standing or recognition. Nothing better helps to explain our situation, the backwardness of our professional rank, than the fact that practically all the publications supposedly devoted to our interests, to the promulgation of our science, are controlled by supply houses. *The standing of a profession is determined by its literature.* It was not until the English physicians were represented by the *Lancet* that they obtained recognition as a class. That the dentists of America, in spite of the manifest progress they have made, in spite of the really remarkable achievements that have been theirs, in spite of the fact that they number thirty-five thousand, have so little recognition to-day in the affairs of the American Medical Association is due, no doubt, in large measure to the fact that the physicians have such standard publications as the *Medical Record, American Medicine,* the *Journal of the American Medical Association,* and half a dozen other periodicals to represent them, while we have nothing except house organs that neither merit respect nor obtain it. Imagine the medical profession dependent for its literature upon a publication owned and edited by Johnson & Johnson, or by the owners of Listerine, or by Parke, Davis & Co., or by a jobbing house.

If we had a periodical literature of known independence and high standing, a literature that would command *general respect*

and *intelligent attention,* it would serve not only to stimulate the profession as a whole, but it would be certain to develop original research work, in which we are now wofully backward. No important research work has ever been accomplished in any division of science, unless it has had the active good will and co-operation of a considerable body of men. And this can only be brought about by the existence of a medium that is in a position to encourage and promulgate results. No section of medicine stands in greater need of the work of the intelligent investigator than ours. We have many problems to solve. In a dozen different directions we are groping in the dark, proceeding along lines where all is speculation, nothing fact. We know little or nothing of the causes of decay, or the causes of erosion, of the histologic structure of enamel. We need better filling-material. We need substances to take the place of unsightly gold. We need light on the best methods of porcelain inlay. We need instruction and information on a *hundred* different subjects. We need better mechanical appliances, and, *above all,* we need a broader spirit, a spirit that will keep men who *perfect* such processes or appliances from running straight to the patent office with them. Such a broader spirit can be fostered only through a broader literature.

What do you suppose would happen to a physician in good standing who should perfect a new instrument or a new process, and straightway secure a patent that would restrict its use? He would be *pilloried* in *his press,* and treated with *contempt* by his associates. But with us it is a matter of course that a man who finds a new way of doing things should at once take the commercial view and get out of it all he can. Have we not all of us seen reputable dentists buzzing about a convention, buttonholing their fellows to buy this thing or that, which they have just perfected and patented? A pitiful and humiliating sight, truly, but unfortunately a common one.

There is, too, another side to this mercenary, patent-seeking spirit that the present conditions have fostered. This is the way in which the supply combination adapts this professional narrowness to its own uses. Its agents in the field, and its representatives in Washington, watch narrowly for new devices, and anything that promises at all is bought up as soon as the patent rights are perfected, or even before. And then the new device is shelved in order that it may not interfere with the stock designs already on the market. No one knows even remotely how many hundred appliances that would make far better work and easier have been shelved this way, with a consequent arrest of development in the science, and a general lowering of the moral tone.

To overcome this hampering spirit in our ranks will require a strong propaganda, and for this there is only one agency on which we may, with any degree of certainty, rely. That is a sound, far-reaching literature that will preach *higher professional standards* and hold the fraternity *up* to them. Manifestly it is idle to look to the house organ for such work. It could not, even if its editors had the inclination, work against the interests of its owners, as it would have to, in order to do any good along these lines. For such work we must look to *independent publications,* conducted in the interests of the workers in the profession, and *not* in the interest of any supply dealer or manufacturer, no matter how rich or how important.

As the standing of a profession is always determined by the character of its literature, so the rise of the members of the profession, in an ethical and worldly sense, may be distinctly traced in the rise of their periodicals. As I have already pointed out, the physicians of England had neither repute nor standing until the *Lancet* made *both* for them. They were merely leeches and ranked with the barbers.

Railroading, both in England and America, was a trade until the founding of distinctive class publications. Similarly, the electrician was an ordinary handicraftsman, until there appeared in this field papers worthy of the new industry. Then electricity became, as it is to-day, one of the most important and dignified professions in industry. Throughout the field of human endeavor similar progress may be traced. A worthy, dignified literature was always the precursor of a worthy, dignified standing. And it will be so with us. We can have neither hope nor promise of real solidity, we can have neither the high rank nor high reputation we merit, until we put forth publications that shall be untrammelled by commercial interests, and that shall be conducted in the interests of the profession and not in the interests of this house or that.

You may rest assured that until there *are* representative publications that shall record progress, stimulate research, and bring about an interchange of ideas, we will advance, if at all, only at an unsatisfactory pace. Indeed, I am not at all certain that, unless we have such a stimulus, we shall advance at all beyond a certain point; that we shall not be overtaken with dry-rot; that the spirit of dwarfing commercialism which rests on us now, will not eventually cause a retrograde, instead of a forward, movement. As it is now, every step in advance that we have taken has been due to the effort of some energetic, public-spirited individual who has been carried forward by a true love of the profession. Unfortunately, there is not enough of this spirit to warrant our relying on it for general and continued advancement.

At any rate, the time has passed when we *ought* to depend upon *disconnected efforts* here and there. We ought to get together in some manner and through some agency and inaugurate a systematic method of stimulating scientific work. We ought to get the profession farther away from the mechanical and commercial side. We have plenty of men in our ranks who would be only too glad to take up the scientific work so urgently needed, if they were assured practical and substantial encouragement. I can name over a dozen men who have in them the capacity to develop this side of our profession. Like all true scientists they are *impractical* in the business sense. We ought to get behind these men, to establish them in a laboratory of research, and provide them with a publication that would enable them to spread before the profession the results of their work.

Unless we do something of this sort, unless we found a proper literature and arrange for a proper system of original research, we will wake up some morning and find the "pre-eminence of the American dentist" a thing of the past. Even now the foreigners are leading us on the purely scientific side of our work, in the development of those principles and ideas that after all must be the foundation of the profession, as against the mere utilitarian foundation of a trade or industry. On our own part, we have grown to rest too well content upon our superior mechanical ability, oblivious of the fact, apparently, that by neglecting the scientific side of the work, we are pushing *away* from the goal instead of toward it.

The laboratories abroad are going deeply into the questions that we have merely touched on. They are digging to the roots of things. They are creating what must always be the true basis of medical work—they are seeking the *cause* of things for which we simply seek the cure. We are, for the most part, simply meeting the difficulties as we come upon them, and attempt remedying them by mechanical processes, instead of going deeper and preventing the difficulties in the first instance. As the result of such a superficial course, we have already begun to lose standing, so that while our representatives who have gone abroad are accorded a high place as operators, we are scoffed at as scientists, and most of the scientific progress that is made is coming out of European laboratories.

This state of things is to be particularly deplored in view of the fact that our profession has always been shown the greatest consideration by American people and American institutions. The National and the State governments have given us every protection and are prepared to foster our work along any line we may desire to push it. But, instead of taking advantage of this condition, we are puttering, making pretty speeches, developing the purely mechanical side of our work, and doing nothing practical or permanent to develop the scientific or fundamental side.

Let us "cut out" of our meetings some of the long-winded papers that merely kill time and interest. Cut out the rehashes of primary facts that have been drilled into college students, and substitute in their place a measure of new scientific work; something that will be as helpful in the *theory* of dentistry as the clinics are in its *practice*.

In conclusion, I would make these suggestions:

*First.*—That we institute some organized measure to prevent the exploitation of our students at the outset of their careers by the supply dealers and manufacturers.

*Second.*—That we keep our meetings and conventions clear of the commercial element.

*Third.*—That we organize a movement that shall bring about the foundation of an independent literature.

*Fourth.*—That we organize a systematic movement for original research.

When we accomplish these *four things*, we will have taken a long step toward the emancipation of our profession from those conditions that have kept us behind all the other professions in the upward movement; we will have done that which will insure the up-lifting of our calling toward the high place it ought to occupy; we will have made it clear that we stand for pure ideals, the advance of science, and the eradication of commercialism. Let us but work together in these matters, and we will cast down the last stone in the wall that divides us from equality with the best there is in medicine. This, gentlemen, is what I mean by "Professional Emancipation and how it may be effected."—*Dental Summary.*

Anæsthesia, Local.—The writer describes his method of producing local anæsthesia. He uses B-eucaine, which is far less dangerous than cocaine, while possessing analgesic properties little, if at all, inferior to it, and with the concurrent use of adrenalin for the purpose of securing a retardation of circulation equivalent to constriction of the part, he has removed some of the objections as to the duration of the analgesia, the extent of the area which can be dealt with, and the amount of the toxic drug to be employed. It is necessary to keep within the safe dose of the drug, and to have at our disposal a large enough quantity of the fluid medium to render it possible to spread the analgesic agent over large areas. For ordinary surgical work the following solution is found by the author to answer well: Distilled water, 140 cubic centimetres; B-eucaine, 0.2 grams; sodium chloride, 0.8 grams; 1 in 1000 adrenalin chloride solution, 10 minims. All this quantity of fluid can be used in an ordinary case if necessary, and is quite sufficient for most. Twice as much may be injected without ill results. The duration of the insensibility is secured by the admixture of the adrenalin. Without it sensation is only abolished by eucaine for about fifteen minutes; with it, for from three to four hours. But the analgesia is produced more slowly when adrenalin is employed with the eucaine. It is therefore well before all larger operations to wait some thirty minutes after injection to allow time for the insensibility to become fully developed. After this the effect appears to deepen for a couple of hours. Waiting has another advantage. When eucaine alone is employed the operation must be done at once. The tissues are still in a state of artificial œdema which masks the anatomic details unpleasantly. By adding adrenalin to the eucaine solution and waiting, the artificial œdema has disappeared and details are very clearly seen. Rapid injection is to be avoided; sudden distention of the tissues is disagreeable, if not painful. The fluid should not be used cold nor too hot, for the same reason. All dragging on the parts is to be avoided, lest structures be pulled upon which lie beyond the area of infiltration. The writer has never seen any depressing effects follow the use of

B-eucaine in a long series of operations. A list of operations done under B-eucaine analgesia is appended, and among these are the following: Abdominal sections, hernia operations, amputations, orchidectomy, removal of cyst of thyroid, removal of silver wire from around the patella, operations for fistula in ano, varicose veins, hydrocele, varicocele, etc.—A. E. Barker (*British Medical Journal,* December 24, 1904.

